# Group B Streptococcal Colonization, Molecular Characteristics, and Epidemiology

**DOI:** 10.3389/fmicb.2018.00437

**Published:** 2018-03-14

**Authors:** Sarah Shabayek, Barbara Spellerberg

**Affiliations:** ^1^Department of Microbiology and Immunology, Faculty of Pharmacy, Suez Canal University, Ismailia, Egypt; ^2^Institute of Medical Microbiology and Hygiene, University of Ulm, Ulm, Germany

**Keywords:** *Streptococcus agalactiae*, colonization, molecular characteristics, epidemiology, serotype

## Abstract

*Streptococcus agalactiae* or group B streptococcus (GBS) is a leading cause of serious neonatal infections. GBS is an opportunistic commensal constituting a part of the intestinal and vaginal physiologic flora and maternal colonization is the principal route of GBS transmission. GBS is a pathobiont that converts from the asymptomatic mucosal carriage state to a major bacterial pathogen causing severe invasive infections. At present, as many as 10 serotypes (Ia, Ib, and II–IX) are recognized. The aim of the current review is to shed new light on the latest epidemiological data and clonal distribution of GBS in addition to discussing the most important colonization determinants at a molecular level. The distribution and predominance of certain serotypes is susceptible to variations and can change over time. With the availability of multilocus sequence typing scheme (MLST) data, it became clear that GBS strains of certain clonal complexes possess a higher potential to cause invasive disease, while other harbor mainly colonizing strains. Colonization and persistence in different host niches is dependent on the adherence capacity of GBS to host cells and tissues. Bacterial biofilms represent well-known virulence factors with a vital role in persistence and chronic infections. In addition, GBS colonization, persistence, translocation, and invasion of host barriers are largely dependent on their adherence abilities to host cells and extracellular matrix proteins (ECM). Major adhesins mediating GBS interaction with host cells include the fibrinogen-binding proteins (Fbs), the laminin-binding protein (Lmb), the group B streptococcal C5a peptidase (ScpB), the streptococcal fibronectin binding protein A (SfbA), the GBS immunogenic bacterial adhesin (BibA), and the hypervirulent adhesin (HvgA). These adhesins facilitate persistent and intimate contacts between the bacterial cell and the host, while global virulence regulators play a major role in the transition to invasive infections. This review combines for first time epidemiological data with data on adherence and colonization for GBS. Investigating the epidemiology along with understanding the determinants of mucosal colonization and the development of invasive disease at a molecular level is therefore important for the development of strategies to prevent invasive GBS disease worldwide.

## Introduction

*Streptococcus agalactiae* or group B streptococcus (GBS) is a pathobiont that is often part of the normal microbiota found in the gastrointestinal and genitourinary tracts of healthy women (Verani et al., 2010). It can cause serious neonatal infections and adult infections. During the early 1930s, GBS was initially identified as a veterinary pathogen and a frequent source of bovine mastitis (Keefe, 1997). The first reported cases of fatal human GBS infections were investigated by Fry (1938). Severe perinatal GBS infections were originally described in the 1960s (Hood et al., 1961). Shortly afterward since the 1970s, GBS emerged as a leading cause of neonatal mortality and morbidity in the United States (Dermer et al., 2004).

The gastrointestinal tract is recognized as a reservoir for GBS and represents most probably the source of vaginal colonization (Meyn et al., 2009). Maternal colonization is the principal route of GBS transmission in early-onset infections as bacteria can spread either *in utero* by ascending infection or during birth through neonatal aspiration of contaminated amniotic or vaginal fluids (Maisey et al., 2008a; Rajagopal, 2009; Verani et al., 2010). About 30–70% of colonized mothers deliver GBS colonized newborns and 1–2% of these develop early-onset infections where heavy colonized mothers are more likely to transmit GBS to their offspring (Anthony et al., 1979; Barcaite et al., 2008; Melin, 2011; Melin and Efstratiou, 2013). However, the route for GBS acquisition in late-onset infections is less clear. It may develop through vertical transmission from mother to neonate, nosocomial transmission, contaminated breast milk or prematurity (Rajagopal, 2009; Le Doare and Kampmann, 2014; Zimmermann et al., 2017).

Group B streptococcus diseases in neonates which develop within the 1st week after birth are designated as early-onset disease (EOD). Late-onset infections (LOD) develop between the 7th day of birth and 2 or 3 months of age. Early-onset infections usually manifest as pneumonia and sepsis while meningitis is most common as a Late-onset event (Verani et al., 2010; Melin and Efstratiou, 2013). Newborns with EOD frequently suffer from respiratory failure which rapidly develops into bacteremia and septic shock. Infants surviving LOD meningitis will develop chronic neurologic sequelae including seizures, cognitive impairment, hearing loss and blindness in up to 50% (Schuchat, 1998; Maisey et al., 2008a; Libster et al., 2012; Melin and Efstratiou, 2013).

During the mid-1990s, the American College of Obstetricians and Gynecologists (ACOG), Centers for Disease Control and Prevention (CDC) and the American Academy of Pediatrics (AAP) recommended intrapartum antibiotic prophylaxis (IAP) to prevent perinatal GBS disease (Schuchat, 1998). This was followed by revised guidelines for the prevention of GBS disease issued in 2002 and the updated guidelines in 2010 (Verani et al., 2010; Schrag and Verani, 2013) which are currently in use. These recommendations included a universal culture-based screening for pregnant women at 35–37 weeks of pregnancy in order to limit IAP to a certain risk group. Widespread active implementation of an IAP program in the United States resulted in an outstanding decrease in the incidence of the disease. Invasive EOD GBS infections declined from 1.8 cases/1000 live births in the early 1990s to 0.26 cases/1000 live births in 2010 (Schrag and Verani, 2013). However, IAP had no impact on late-onset infections and thus LOD GBS diseases continue as the leading cause of neonatal morbidity and mortality (Verani et al., 2010; Schrag and Verani, 2013).

Asymptomatic GBS carriage is frequent and in general harmless in healthy women. GBS can, however, cause serious infections in pregnant women. Invasive maternal illness due to GBS in pregnant and postpartum women include bloodstream infections, meningitis, osteomyelitis, and endocarditis. Non-invasive maternal diseases manifest as bacteriuria, amnionitis, fasciitis, cellulitis, endometritis and wound infections associated with episiotomies or cesarean deliveries. In addition, GBS has been increasingly reported as being responsible for invasive disease in elderly and immunocompromised patients. In non-pregnant adults, GBS diseases include arthritis, endocarditis, pneumonia, bacteremia and urinary tract infections, as well as soft tissue, skin, and bone infections. Susceptibility to GBS is increased in the elderly and immunocompromised individuals with underlying conditions such as diabetes, cancer, and HIV (Schuchat, 1998; Farley, 2001; Phares et al., 2008; Sendi et al., 2008; Skoff et al., 2009). Overall neonatal as well as adult infections are, to a large percentage, endogenous infections with colonization of gastrointestinal and vaginal mucosal surfaces as a first crucial step. Nevertheless, in many cases, GBS persists as a commensal bacterium of the microbiota and does not proceed to cause invasive infections. During the last decades, hypervirulent clones have been identified and GBS regulators have been characterized that control the expression of major virulence factors, however, the switch from commensal to invasive pathogen is still incompletely deciphered. Understanding the determinants of GBS mucosal colonization as well as the different stages of the pathobiont lifestyle at a molecular level is therefore important for the development of prevention strategies to control invasive GBS disease. This review combines for first time epidemiological data with data on adherence and colonization for GBS. It covers the following aspects: GBS colonization rates and serotype distribution in different countries and the molecular epidemiology of GBS colonization, determined through MLST sequence types. Adherence properties of GBS will be summarized under the following headings, biofilm formation, and GBS colonization and adherence at a molecular level, which is be subdivided into (i) GBS adhesins to extracellular matrix proteins (ECM), (ii) GBS adhesins to cellular targets, and (iii) GBS pili. The last two sections describe the transition of GBS from commensal to pathogen and current efforts on immunoprophylaxis.

## GBS Colonization in Different Countries: Colonization Rates and Serotype Distribution

Since vaginal GBS colonization represents the most important risk factor for the development of invasive neonatal infections, colonization rates have been investigated in many different countries. Interestingly the GBS colonization rate among pregnant women in vagina and/or rectum is quite similar worldwide with some variations ranging from 10 to 30% in United States, 6.5% up to 36% in Europe, 7.1 to 16% in Asia, 9.1 to 25.3% in the Middle East, and 11.9 to 31.6% in Africa (Schuchat, 1998; Barcaite et al., 2008; Ippolito et al., 2010; Verani et al., 2010). The GBS colonization status, however, is intermittent and can be transient during pregnancy. Positive colonizers in early or mid-pregnancy may turn into negative colonizers at delivery (Hansen et al., 2004; Verani et al., 2010). Hence, timing of GBS screening and specimen collection is important to accurately predict colonization status at delivery. According to the CDC guidelines, the intrapartum colonization status is best determined in the late third trimester at no more than 5 weeks before delivery (Verani et al., 2010).

While GBS colonization rates may appear quite similar in different regions of the world, serotype prevalence and distribution is geographically distinct. Serotype classification is based on a sialic acid-rich capsular polysaccharide (CPS) which is one of the most important virulence factors in GBS that is involved in GBS persistence and survival within the host. CPS plays a critical role in immune evasion through its mimicry with the host carbohydrate epitopes. It also interferes with the complement-dependent defense pathways, dampens the phagocytic function of neutrophils and facilitates bacterial internalization and intracellular survival inside dendritic cells. Moreover, CPS has been reported to mediate biofilm formation of GBS in the presence of human plasma (Lemire et al., 2012; Pezzicoli et al., 2012; Chang et al., 2014a,b; Xia et al., 2015). At present, as many as 10 serotypes (Ia, Ib, and II–IX) are recognized (Slotved et al., 2007; Le Doare and Heath, 2013). The distribution and predominance of certain serotypes is susceptible to variations and can change over time. Serotypes Ia, Ib, II, III, and V are prevalent colonizers in the United States and Europe (Johri et al., 2006; Ippolito et al., 2010; Lamagni et al., 2013; Melin and Efstratiou, 2013; Florindo et al., 2014; Fabbrini et al., 2016). Serotypes VI and VIII are the most prevalent among pregnant women in Japan (Lachenauer et al., 1999; Matsubara et al., 2002) while serotypes IV and V predominate in the United Arab Emirates and Egypt, respectively (Amin et al., 2002; Shabayek et al., 2014). Serotype Ia is common in Mexico and is consistently higher in North America in comparison to other areas (Ippolito et al., 2010). The most recently characterized novel GBS serotype IX was reported from Denmark (Slotved et al., 2007). The geographical distribution of the most prevalent colonizing serotypes is shown in **Table 1**. On the other hand, serotype III is the most dominant invasive clone accounting for the majority of late-onset meningitis cases in neonates (Lamy et al., 2006; Tazi et al., 2010; Bellais et al., 2012; Florindo et al., 2014; Alhhazmi et al., 2016) while serotype Ia and V are dominant invasive isolates in non-pregnant cases (Phares et al., 2008; Alhhazmi et al., 2016). However, recent studies indicate the emergence of invasive serotype IV strains in neonates and adults (Ferrieri et al., 2013; Teatero et al., 2015; Alhhazmi et al., 2016) and among colonizing strains (Diedrick et al., 2010). A similar scenario could be expected for serotypes VI and VIII which are rarely reported outside Japan. In Malaysia, serotype VI was found among the dominant colonizing strains and the second most common isolate in adults with skin and soft tissue infections due to GBS (Karunakaran et al., 2009; Dhanoa et al., 2010). In Egypt, serotype VI was reported as a common colonizing serotype in women (Shabayek et al., 2014). And recently, Lin et al. (2016) reported a clonal dissemination of serotype VI among colonizing and invasive isolate in central Taiwan. Likewise, several studies identified sporadic strains of serotype VIII both as colonizing strains and as causative agents for invasive GBS disease (Paoletti et al., 1999; Hoshina et al., 2002; Ekelund et al., 2003; Karunakaran et al., 2009; Ippolito et al., 2010; Alhhazmi et al., 2016). Similar serotype emergence was earlier reported for serotype V in the United States and in Europe (Hickman et al., 1999; Flores et al., 2015). An increasing diversity of GBS serotypes, as well as serotype switching, represent powerful immune evasion strategies that may severely impair vaccine efforts, that currently rely on a conjugate vaccine incorporating a limited number of GBS serotypes. A close monitoring of the changing serotype distribution occurring in many different countries is therefore crucial in guiding GBS vaccination development.

**Table 1:** Geographical distribution of the most prevalent colonizing group B streptococcus (GBS) serotypes.

Most prevalent colonizing serotype(s)	Region	Reference
Ia, Ib, II, III, V	North America, United States, Europe	Johri et al., 2006; Ippolito et al., 2010; Lamagni et al., 2013; Melin and Efstratiou, 2013; Florindo et al., 2014; Fabbrini et al., 2016
Ia	Mexico	Ippolito et al., 2010
IV, Ia	United Arab Emirates	Amin et al., 2002
V	Egypt	Shabayek et al., 2014
Ia, VI	Malaysia	Karunakaran et al., 2009; Dhanoa et al., 2010
VI, VIII	Japan	Lachenauer et al., 1999; Matsubara et al., 2002

## Epidemiology of GBS Colonization: MLST Sequence Types

Capsular serotyping is the traditional tool used in GBS classification for epidemiological purposes. However, in the era of molecular epidemiology with the availability of numerous bacterial genomes and novel nucleotide sequencing-based tools our traditional picture of GBS epidemiology changed. In respect to corresponding genomes, gene content does not necessarily correlate with the assigned serotype. In 2003, a seven-gene multilocus sequence typing scheme (MLST) was introduced for GBS classification. First studies employing this MLST scheme revealed that capsular serotype is not restricted to a particular MLST sequence type (ST) and GBS strains with the same ST may have different serotypes (Jones et al., 2003). Several STs are grouped into clonal complexes (CC) when sharing six or seven matching alleles. The number of a particular CCs is designated after its ancestor ST or the predominant ST within this clone (Luan et al., 2005). By MLST the vast majority of the human GBS isolates can be grouped into six CCs which are CC1, CC10, CC17, CC19, CC23, and CC26 (Sørensen et al., 2010; Da Cunha et al., 2014). Interestingly, most of the bovine strains were exclusively designated as subtypes of the CC61 and CC67 which have never been reported for any of the human strains (Bisharat et al., 2004; Sukhnanand et al., 2005; Sørensen et al., 2010; Yang et al., 2013; Springman et al., 2014).

With the availability of MLST data, it became clear that GBS strains of certain CCs possess a higher potential to cause invasive disease, while other harbor mainly colonizing strains. MLST for a global GBS collection identified STs 1 and 19 to be significantly more associated with asymptomatic colonization and ST-23 was common for carriage and invasive GBS (Jones et al., 2003). This is in agreement with a study (Manning et al., 2008) that reported STs 1, 19, and 23 as the predominant colonizers in pregnant women and identified them as being well adapted to the vaginal mucosa with a poor invasion ability. Consistently, Teatero et al. (2017) found STs 1 and 23 the most frequent clones in colonized pregnant women. In general, the dominance of strains belonging to CCs 1, 23, and 19 among asymptomatic pregnant women was consistently reported (Jones et al., 2003; Luan et al., 2005; Springman et al., 2014), indicating that for these CCs being a mucosal commensal predominates.

A major clone responsible for a large proportion of invasive neonatal infections are the CC17 strains mostly belonging to serotype III. ST-17 strains first gained special interest due to their strong association with neonatal meningitis. Strains belonging to the CC17 are reported to be hypervirulent accounting for more than 80% of the GBS late-onset neonatal infections and are often but not exclusively associated with meningitis (Jones et al., 2003; Lamy et al., 2006; Manning et al., 2009; Tazi et al., 2010; Bellais et al., 2012; Florindo et al., 2014). Comparative phylogenetic analysis of human and bovine isolates revealed CC17 strains as a homogenous group with a recent origin and limited recombination in comparison to other CCs (Sørensen et al., 2010; Da Cunha et al., 2014). Moreover, evolutionary analysis between GBS isolates of human and bovine origin found ST-17 as the only human lineage that is clustered within the bovine population (Bisharat et al., 2004). The investigation suggested ST-17 to have bovine ancestors (Bisharat et al., 2004), which was supported by another study (Héry-Arnaud et al., 2007) providing evidence for a bovine origin of CC17 by investigating the prevalence of mobile genetic elements. However, a very close connection between the bovine CC67 and the human CC17 was also challenged by a more comprehensive genetic analysis of 238 bovine and human strains (Sørensen et al., 2010). The emergence of a highly virulent GBS clone, causing a large majority of neonatal infections, from a bovine ancestor provides intriguing phylogenetic aspects for placing GBS close to zoonotic diseases. Thus the question of how zoonotic GBS may be is currently controversially discussed and represents an exciting and ongoing scientific question to be solved.

For adult invasive infections, molecular clones other than CC17 are important. A significant percentage of GBS invasive disease in non-pregnant adults is resulting from strains belonging to serotype V (Phares et al., 2008; Lamagni et al., 2013; Teatero et al., 2014; Flores et al., 2015). Molecular epidemiology revealed a remarkable association of ST-1, a subtype of CC1, with serotype V GBS causing invasive disease. ST-1 invasive isolates from adults and neonates are mostly belonging to serotype V (Salloum et al., 2011). A recent analysis of a large cohort of GBS isolates of invasive serotype V strains from adults in United States and Canada (Flores et al., 2015). reported 92% of the serotype V strains as ST-1 whereas the most predominant sequence type among the non-ST-1 clones was ST-19. The authors regard the emergence of ST-1 strains as a leading cause of adult disease in the 1990s. Similar to human ST-17, the human ST-1 is suspected to originate from a bovine ancestor. Whole-genome sequencing recognized a 1992 ST-1 clone to be closely related to a 1970s Swedish strain causing cow mastitis. The ST-1 human GBS were found to possess mutations at loci involved in capsule production, pilus expression, and two-component regulatory systems (Flores et al., 2015) which all have been reported as key virulence factors in GBS (Maisey et al., 2008a; Rajagopal, 2009) and may thus represent the adaptation of this strain to human infections.

Particular interest should also be paid to the increasing emergence of type IV among colonizing and invasive GBS isolates. In several recent investigations, this serotype appears to be a hotspot for genetic recombination events, supported by the detection of serotype IV strains with different genetic backgrounds. Multiple studies demonstrated ST-196 as the most frequent sequence type of serotype IV isolates (Gherardi et al., 2007; Héry-Arnaud et al., 2007; Martins et al., 2007; van der Mee-Marquet et al., 2008; Lartigue et al., 2009; Diedrick et al., 2010). However, other investigations reported the STs 452 and 459, belonging to CCs 23 and 1, respectively, as the predominant colonizing type IV strains and found ST-196 less frequently (Diedrick et al., 2010; Ferrieri et al., 2013; Teatero et al., 2017). Comparing a collection of emerging type IV isolates in United States belonging to two distinct time periods from 1995 to 2000 and from 2004 to 2008, the older strains were more like a 1970s prototype reference strain with similar PFGE profiles whereas the more recent isolates showed PFGE profiles with considerable differences (Diedrick et al., 2010). Independent studies identified the invasive characteristics of type IV strains to be a result of capsular switching in CC17 strains accompanied with the acquisition of the HvgA adhesin, which has initially been isolated from hypervirulent CC17 serotype III strains (Teatero et al., 2014; Alhhazmi et al., 2016). Capsular switching events of CC17 strains have previously been observed (Da Cunha et al., 2014) leading to serotype IV ST-256 lineage.

For colonizing as well as invasive GBS strains dominant serotype-MLST genotypes associations have been observed such as serotype III with ST-17, serotype V with ST-1 and serotype IV with ST-196. Unusual associations are most likely to be due to capsular switching which resulted from recombination events around the capsular locus (Teatero et al., 2017). Investigation of the increase of type IV strains suggested the evolution of hybrid genomes including sequences from hypervirulent ST-17 strains (Teatero et al., 2016). Thus growing evidence points to an ongoing emergence of novel virulent GBS clones requiring continuous epidemiological surveillance.

## Biofilm Formation

Colonization and persistence in different host niches is dependent on the adherence capacity of GBS to host cells and tissues. This then facilitates bacterial cell aggregation and formation of sessile communities known as biofilms. Bacterial biofilms represent well-known virulence factors with a vital role in persistence and chronic infections. In the host environment, bacteria are often protected from the immune system by building sessile colonies embedded in an extracellular matrix of polysaccharides representing the biofilm. For GBS the bacterial capsule and type IIa pili have been demonstrated to play an important role in biofilm formation (Konto-Ghiorghi et al., 2009; Xia et al., 2015). Host environmental conditions are crucial determinants in developing bacterial biofilms (Costerton et al., 1999; Lewis, 2005; Nobbs et al., 2009; Rosini and Margarit, 2015). Contradictory data are available concerning the environmental cues favoring biofilm communities in GBS (Rosini and Margarit, 2015). As a normal inhabitant of the vagina, acidic pH seems to be optimal for GBS colonization. Early investigations reported enhanced GBS adherence to vaginal epithelial cells under low pH in comparison to neutral pH (Zawaneh et al., 1979; Tamura et al., 1994). In line with these observations, a significantly higher biofilm production of colonizing GBS isolates from pregnant women was demonstrated at pH 4.5 vs. pH 7 (Ho et al., 2013). Similarly, enhanced biofilm formation of GBS was shown under acidic pH conditions in comparison to neutral pH with the strongest biofilm producing GBS isolates belonged to the ST-17 sequence type. In respect to GBS origins, higher frequencies of strong biofilm producers were found among neonatal strains in comparison to colonizing strains (D’Urzo et al., 2014). However, a recent investigation reported invasive GBS belonging to CC17 and CC19 lineages as weak biofilm formers while GBS isolated from asymptomatic carriers were found to be strong biofilm producers (Parker et al., 2016). One possible explanation for this discrepancy is the experimental set up of the study since GBS biofilm formation was tested at neutral pH conditions and not under acidic pH. Furthermore, the presence of human plasma was shown to promote GBS biofilm formation (Xia et al., 2015).

In summary, biofilms allow long-term bacterial persistence and protect bacteria from recognition by the immune system. For GBS low pH and the presence of plasma appear as crucial environmental factors through controlling the expression of bacterial surface-associated structures, such as pili and the capsule, which are both involved in promoting bacterial biofilm formation.

## GBS Colonization and Adherence at a Molecular Level

### GBS Adhesins to Extracellular Matrix Proteins

**FIGURE 1:**
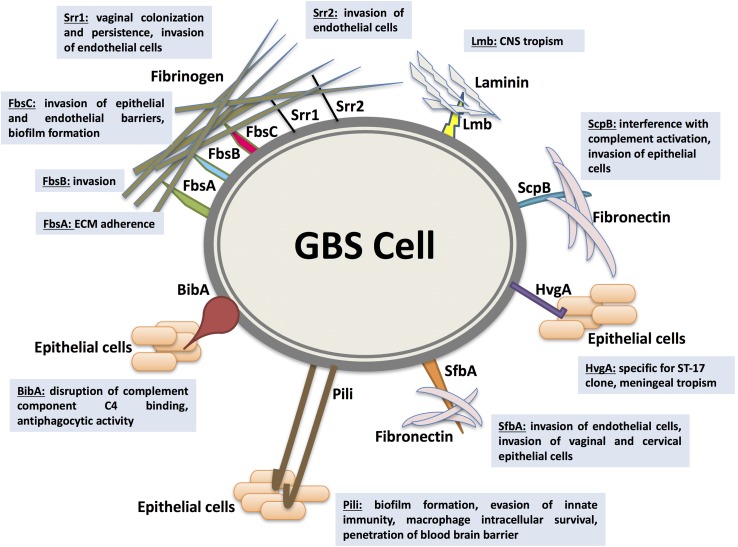
Major adhesins mediating *Streptococcus agalactiae* (GBS) interaction with host cells. GBS colonization, persistence, translocation, and invasion of host barriers are largely dependent on their ability to adhere to host cells and extracellular matrix proteins (ECM), an important step in breaching cellular barriers. The best characterized surface proteins mediating GBS adherence are the fibrinogen-binding proteins Fbs (including FbsA, FbsB, FbsC, or BsaB, the serine-rich repeat glycoproteins Srr1 and Srr2), the laminin-binding protein (Lmb), the Streptococcal C5a peptidase from group B (ScpB), the streptococcal fibronectin-binding protein A (SfbA), and the GBS immunogenic bacterial adhesin BibA. In addition, surface-protruding structures like pili are considered as essential adhesins in promoting GBS colonization, persistence, biofilm production, and central nervous system invasion. Associated virulence traits are illustrated for each adhesin as follows. FbsA was mainly shown to promote adherence whereas FbsB was shown to be required for invading human cells. Srr1 and Srr2 were reported to mediate invasion of microvascular endothelial cells. Additionally, Srr1 was demonstrated to promote vaginal colonization and persistence. FbsC was recently characterized to promote invasion of epithelial and endothelial barriers and biofilm formation. The Lmb adhesin appears to have a pronounced role in bacterial tropism of the central nervous system (CNS). ScpB interrupts complement activation through cleaving the neutrophil chemoattractant C5a. It is also involved in invasion of human epithelial cells. The SfbA adhesin is involved in human brain microvascular endothelial cells invasion. Furthermore, SfbA contributes to GBS invasion of vaginal and cervical epithelial cells and hence may take part in GBS colonization and niche establishment in the vagina. BibA was reported to aid GBS survival in human blood through interfering with the classic complement pathway by binding the C4-binding protein and by conferring anti-phagocytic activity against opsonophagocytic killing by human neutrophils. HvgA is specific for the hypervirulent clone ST-17. It was suggested to promote meningeal tropism in neonates. Pili in GBS have been shown to be primarily involved in epithelial cell colonization, biofilm formation, translocation, and invasion. PI-1 pili were also found to play an important role in evasion of innate immunity mechanism. The PI-2b protein, however, was demonstrated to increase the intracellular survival in macrophage. Pilus 2b was further identified as important for infection and penetration of the blood brain barrier.

As an opportunistic commensal constituting a part of the intestinal and vaginal physiologic flora, GBS colonization, persistence, translocation, and invasion of host barriers are largely dependent on their adherence abilities to host cells and ECM (Singh et al., 2012; Landwehr-Kenzel and Henneke, 2014). Functionally characterized adhesins mediating GBS adherence and/or invasion within the host are the fibrinogen-binding proteins (Fbs), the laminin-binding protein (Lmb), the group B streptococcal C5a peptidase (ScpB), the streptococcal fibronectin-binding protein A (SfbA), and the GBS immunogenic bacterial adhesin (BibA). In addition, surface-protruding structures comprised of multiples genes like pili are considered as essential adhesins in promoting GBS colonization, persistence, biofilm production, and central nervous system invasion. Major adhesins mediating GBS interaction with host cells are depicted in **Figure 1**.

Up to date, five Fbs have been characterized in GBS; FbsA (Schubert et al., 2004), FbsB (Gutekunst et al., 2004), the serine-rich repeat glycoproteins Srr1 and Srr2 (Seo et al., 2012, 2013), and recently FbsC or BsaB (Buscetta et al., 2014; Jiang and Wessels, 2014). In general, invasive GBS isolates display stronger fibrinogen-binding abilities in comparison to colonizing ones (Rosenau et al., 2007). FbsA was mainly shown to promote adherence (Schubert et al., 2004) whereas FbsB was shown to be required for invading human cells (Gutekunst et al., 2004). Srr1 and Srr2 were reported to mediate invasion of microvascular endothelial cells (Seo et al., 2012, 2013). Additionally, Srr1 was demonstrated to promote vaginal colonization and persistence, since a Srr1-deficient mutant displayed reduced persistence in a mouse GBS vaginal colonization model (Sheen et al., 2011). FbsC was recently characterized to promote invasion of epithelial and endothelial barriers. FbsC deletion mutant of GBS displayed a drastic reduction in abilities for adherence, invasion and biofilm formation. Besides, virulence abilities of FbsC deletion mutant were impaired in murine infection models (Buscetta et al., 2014). Interestingly, the fibrinogen-binding abilities of the hypervirulent CC17 clones are mainly attributable to FbsB more than FbsA. Deletion mutants of *fbsB* displayed 78–80% reduction in their binding abilities vs. 49–57% as encountered with *fbsA* deletion mutants of CC17 strains (Al Safadi et al., 2011). Accordingly, the relative transcription level of *fbsB* was up to 12.7-fold higher than *fbsA* gene in CC17 stains (Al Safadi et al., 2011). Moreover, Srr2 was highly expressed and exclusively detected in ST-17, however, Srr1 was absent (Seifert et al., 2006; Seo et al., 2013). Furthermore, CC17 strains are devoid of FbsC. The *fbsC* gene is not adequately expressed in CC17 strains because of a lineage-dependent frameshift mutation (Buscetta et al., 2014).

In addition to the Fbs family, the Lmb adhesin appears to have a pronounced role in bacterial tropism of the central nervous system. Spellerberg et al. (1999) reported Lmb to be essential for GBS colonization of damaged epithelium and subsequent translocation into the bloodstream. This role was later confirmed by Tenenbaum et al. (2007) as they demonstrated mutation of the *lmb* gene to result in a dramatic reduction in GBS invasion of the brain microvascular endothelial cells. In consistence, Al Safadi et al. (2010) displayed higher expression levels of Lmb in GBS strains associated with meningitis in comparison to other isolates whereas the expression levels of other ECM-binding proteins, such as ScpB mediating fibronectin binding ability, remained unchanged.

ScpB or the group B ScpB is a surface associated serine protease that both interrupts complement activation through splitting the neutrophil chemoattractant C5a and mediates bacterial binding to fibronectin (Chmouryguina et al., 1996; Bohnsack et al., 1997; Cheng et al., 2002; Lindahl et al., 2005). The fibronectin binding ability conferred by the *scpB* gene appears to be involved in cellular adherence and invasion. In frame deletion mutation of *scpB* gene significantly reduced invasion of human epithelial cells *in vitro* (Cheng et al., 2002). Strikingly, *scpB* and *lmb* genes were found to be encoded on a composite transposon where the *scpB* gene is positioned directly upstream of the *lmb* gene. The *scpB-lmb* intergenic region has been described as a hot spot for integration of the GBS mobile genetic elements GBSil and IS*1548* which are located in the promoter region of the *lmb* gene (Franken et al., 2001; Granlund et al., 2001; Luan et al., 2003, 2005; Broker and Spellerberg, 2004). Al Safadi et al. (2010) reported a marked increase in the transcription levels of *lmb* gene for invasive GBS isolates carrying IS*1548* in the *scpB-lmb* intergenic region associated with an increased laminin binding ability. However, no influence was observed on the *scpB* gene. Deletion mutation of IS*1548* revealed IS*1548* to act as an *lmb* gene up-regulator when compared to the wild-type parent strains. Interestingly the ability of GBS to colonize human mucosal surfaces seems to be closely linked to the presence of this composite transposon carrying *scpB* and *lmb*. In a large percentage of bovine strains, the encoded genes are absent while the presence in human colonizing strains, as well as invasive strains, is close to 100% (Franken et al., 2001; Sørensen et al., 2010; Rato et al., 2013).

More recently, a novel GBS fibronectin binding protein has been identified (Mu et al., 2014). It was designated as streptococcal fibronectin-binding protein A (SfbA) and reported to be highly conserved in GBS mediating cellular invasion but not adherence. SfbA was shown to be directly involved in fibronectin binding and human brain microvascular endothelial cells invasion. When expressed in recombinant non-pathogenic *Lactococcus lactis*, fibronectin binding ability was significantly greater in comparison to a SfbA negative control strain. The investigation also demonstrated SfbA to be primarily involved in brain microvascular endothelial cells invasion. Infection of mice with *sfbA* mutants resulted in a reduced ability to breach the blood brain barrier and subsequent meningitis. This is supported by a study showing SfbA to be crucial for invasion of astrocytes which are physically associated with the brain endothelial cells (Stoner et al., 2015). Furthermore, SfbA contributes to GBS invasion of vaginal and cervical epithelial cells and hence may take part in GBS colonization and niche establishment in the vagina (Mu et al., 2014). Another fibronectin binding protein was described in 2014 (Jiang and Wessels, 2014). BsaB or the bacterial surface adhesin of GBS is a fibronectin and laminin-binding protein which is involved in GBS binding to epithelial cells and in biofilm formation. Deletion of *bsaB* gene and a cotranscribed upstream region significantly abrogated GBS adherence to VK2 vaginal epithelial cells *in vitro* and immobilized fibronectin. However, genome and sequence analysis revealed BsaB and FbsC as identical proteins encoded by the same gene (Buscetta et al., 2014). The obtained results are in agreement with Jiang and Wessels (2014), except that FbsC or BsaB was found to mediate GBS attachment to fibrinogen instead of fibronectin. Hence, BsaB was renamed to FbsC.

The multitude of GBS adhesins allowing attachment to different ECM, stresses the importance of this step in GBS pathogenesis, which was confirmed in different *in vivo* models. In this regard, fibrinogen binding may play an especially important role as demonstrated by the presence of numerous fibrinogen binding proteins. These may represent a kind of “backup” system in cases where the primary fibrinogen adhesin was rendered non-functional.

### GBS Adhesins to Cellular Targets

Besides adherence to ECM, the adhesion to host cells plays an important role in the pathogenesis of GBS. An essential adhesin in this context is the GBS immunogenic bacterial adhesin (BibA). It is a cell wall-anchored protein which is well-conserved in GBS and is involved in bacterial binding to human epithelial cells (Santi et al., 2007, 2009b). A knockout mutant displayed impaired adherence capacity to the lung, intestinal, and cervical epithelial cells (Santi et al., 2007). Overexpression of BibA resulted in increased adherence to human epithelial cells in recombinant wild-type strains harboring a *bibA* plasmid (Santi et al., 2007). In addition, BibA was reported to aid GBS survival in human blood through interfering with the classic complement pathway by binding the C4-binding protein and by conferring anti-phagocytic activity against opsonophagocytic killing by human neutrophils (Santi et al., 2007, 2009b). A total of four variants of BibA (I, II, III, and IV) were described in GBS (Brochet et al., 2006; Santi et al., 2007, 2009b). Interestingly, variant IV, which was found to be highly similar to the bovine BibA counterparts, was exclusively associated with ST-17 strains (Lamy et al., 2006; Santi et al., 2009b). Thus, BibA seems to be a multifactorial virulence factor in regard to GBS as a pathobiont. It contributes to GBS mucosal colonization and adherence to host cells and then confers resistance to phagocytic killing at a stage when the switch to invasive GBS infection has occurred.

The GBS hypervirulent adhesin (HvgA) is a novel cell wall anchored protein that is specific for the hypervirulent clone ST-17. It was first described (Tazi et al., 2010) as being strongly associated with ST-17 causing neonatal meningitis in LOD. It was suggested to promote meningeal tropism in neonates through efficient intestinal colonization and subsequent translocation across the intestinal and the blood brain barriers. Bypassing intestinal colonization by intravenous infection resulted in a significant decrease in the amount of bacteria reaching the central nervous system. HvgA was required for intestinal colonization in orally infected mice for meningitis development. In addition, HvgA was found to mediate GBS adherence to intestinal epithelial cells, choroid epithelial cells, and microvascular endothelial cells (Tazi et al., 2010). Clones expressing HvgA exhibited greater adherence abilities than non-expressing ones. HvgA thus contributes to colonization as well as invasion of hypervirulent clones (Tazi et al., 2010).

### GBS Pili

Further structures that are crucial for GBS adhesion are pili. Different from their Gram-negative counterparts, pili in GBS have been shown to be primarily involved in epithelial cell colonization, biofilm formation, translocation, and invasion. Pili are cell-wall anchored appendages extending from the bacterial surface. They contain covalently linked multimeric motifs that are composed of three pilin proteins, the pilus shaft backbone protein (BP) or PilB subunits, and the two ancillary proteins AP1, AP2 located at the pilus tip (PilA subunit, the pilus-associated adhesin) and pilus base (PilC subunit, the pilus anchor), respectively, (Dramsi et al., 2006; Rosini et al., 2006; Maisey et al., 2007, 2008b; Cozzi et al., 2015). While PilB has been shown to be involved in bacterial invasion and paracellular translocation mediating resistance to phagocytic killing and virulence, PilA was found contributing to cellular adherence and colonization (Dramsi et al., 2006; Krishnan et al., 2007; Maisey et al., 2007, 2008b; Pezzicoli et al., 2008; Sheen et al., 2011). Three pilus variants named PI-1, PI-2a, PI-2b were reported in GBS representing two pilus islands (PI) where PI-2a and PI-2b are variants of the pilus island 2 (PI-2). All characterized GBS strains harbored at least one variant or a combination of two pilus islands (Rosini et al., 2006; Margarit et al., 2009; Springman et al., 2014). PI-1 pili were also found to play an important role in evasion of innate immunity mechanism. They diminished macrophage-mediated phagocytic killing of GBS by 50% with no influence on complement-promoted opsonophagocytic killing by neutrophils (Jiang et al., 2012). Strikingly, PI-1 pili do not appear to contribute to bacterial adhesion to lung, vaginal or cervical epithelial cells (Jiang et al., 2012). The PI-2a pili were found to have a specific involvement in adherence and biofilm formation and not PI or PI-2b (Konto-Ghiorghi et al., 2009; Rinaudo et al., 2010). The PI-2b protein, however, was demonstrated to increase the intracellular survival in macrophage (Chattopadhyay et al., 2011). In addition, a special role for pilus type 2b has been suggested in promoting strain invasiveness and bacterial host cell interactions. Mutants of pilus 2b possess less adherence and invasion capacities for epithelial and endothelial cells (Lazzarin et al., 2017). Pilus 2b was further identified as important for infection and penetration of the blood brain barrier. These results are supported by an investigation of the distribution of pilus islands among GBS strains belonging to ST lineages of human and bovine origin (Springman et al., 2014). In addition, the distribution of pili islands appears to determine the capacity for colonization or invasive infections. Invasive GBS were more likely to carry a combination of PI and one of the PI-2 variants in comparison to maternal colonizing isolates. Moreover, GBS causing invasive neonatal disease including all CC-17 strains were harboring PI-1 plus PI-2b. Earlier genomic studies showed pilus type 2b to be conserved in the ST-17 hypervirulent clone (Brochet et al., 2006). Interestingly PI-2b pilus variants are almost exclusively present in bovine GBS isolates. These bovine strains mostly lack PI-1, unlike the human isolates which commonly encode the pilus PI-1 in association with one of the PI-2 variants (Springman et al., 2014).

## GBS Transition From Commensal to Pathogen

Group B streptococcus is a commensal bacterium of the vagina and the gastrointestinal tract of healthy adults. However, as a pathobiont, GBS can convert from the asymptomatic mucosal carriage state to a bacterial pathogen causing invasive infections. For neonatal infections that implies a switch from bacterial survival in the acidic host environment of the vagina to resistance of the immune system in the pH neutral environment of human blood. To address bacterial adaptation to these environmental changes, a genome-wide transcription analysis was performed in order to investigate GBS responses to acid stress in comparison to neutral pH conditions (Santi et al., 2009a). This study demonstrated the modulation of genes involved in GBS adaptation and vaginal persistence as well as virulence-related genes in response to fluctuating environmental pH which are mostly under the control of the CovRS two-component regulatory system (TCS). Among the adherence factors that were demonstrated to be under the control of CovRS are Fbs proteins, Lmb, C5a, BibA, HvgA, and pili (Lamy et al., 2004; Jiang et al., 2008, 2012; Santi et al., 2009a; Tazi et al., 2010; Park et al., 2012). These observations suggested that GBS translocation from acidic to neutral niches switches on virulence-related genes, favoring the transition from commensal to invasive bacterial pathogen. The CovRS system is a well-studied TCS in GBS and a major virulence regulator. The contribution of CovRS in coordinating GBS gene expression and virulence has been investigated in many studies (Lamy et al., 2004; Jiang et al., 2005, 2008; Di Palo et al., 2013; Faralla et al., 2014). Patras et al. (2013) conducted a global transcription profiling of human vaginal epithelial cells exposed to a CovR-deficient mutant in comparison to the wild-type strain and studied a mouse model of GBS vaginal colonization. Their major observation was that loss of CovR signaling promoted exaggerated host inflammatory reactions against GBS infection. Hence, the authors concluded that CovRS control of GBS virulence gene expression is crucial in maintaining GBS vaginal colonization by avoiding host immune responses (Patras et al., 2013). Apart from regulation through TCS, GBS genomic adaptations seem to take place during GBS transition from commensal to pathogen. Comparing the whole genomes of GBS pairs from infected newborns and their mothers genomic mutations were found with critical phenotypic impacts, including CovR mutations (Almeida et al., 2015). Genomic adaptations may also impact dynamics of maternal GBS colonization before and after delivery. Alterations in STs and serotypes can be observed in 18.3% of pregnant women who were positive colonizers before and after delivery (Manning et al., 2008). Definite STs appear to be related with GBS clearance and persistence. Not surprisingly capsule expression is linked to invasion capabilities of GBS. A significantly higher proportion of non-typeable strains are present among colonizing isolates in comparison to invasive isolates (Teatero et al., 2017). This may indicate that persistent colonizers undergo several lateral DNA exchanges leading to the abolishment of capsule expression since encapsulation is less important during carriage. Capsule loss has previously been suggested to favor GBS colonization and diminish virulence (Rubens et al., 1987) and capsule production is also regulated through CovRS. Thus the results of multiple independent investigations strengthen the role of CovRS as a central GBS regulator controlling the switch from mucosal colonization to invasive infection.

## Immunoprophylaxis

The ability of GBS to cause neonatal invasive infections is dependent on the maternal antibody titer. Early work done by Rebecca Lancefield in the 1930s (Lancefield, 1938) reported protection against GBS infection in mice by CPS-specific antiserum in rabbits. Baker and Kasper (1976) demonstrated an inverse association between the levels of maternal serotype-specific capsular antibodies and the increased susceptibility to invasive GBS disease in newborns. This association was confirmed in later investigations (Lin et al., 2001, 2004; Baker et al., 2014). Vaccination seems, therefore, to be a good alternative to peripartal antibiotic prophylaxis in order to prevent neonatal GBS infections. However, no licensed vaccines are available (Verani et al., 2010; Madhi and Dangor, 2017). The sialylated CPS has been the main target for GBS vaccine in most clinical trials. Phases I and II clinical trials demonstrated the safety and immunogenicity of monovalent CPS-conjugate vaccines among healthy women (Paoletti and Kasper, 2003). However, monovalent formulations with serotype-specific immune responses are not sufficient to provide protection against the different GBS serotypes found in invasive infections. Besides, possible capsular switching forced by the selective pressure exerted due to GBS monovalent vaccines can be expected. Multivalent formulations would provide broader vaccine coverage and overcome problems of serotype switching (Johri et al., 2006; Brueggemann et al., 2007; Nuccitelli et al., 2011; Heath et al., 2017). The Safety and immunogenicity of a trivalent GBS conjugate vaccine based on serotypes Ia, Ib, and III have has been evaluated in Phases I and II trials and there are considerations for a Phase III trial (Madhi et al., 2013; Donders et al., 2016; Heyderman et al., 2016; Kobayashi et al., 2016a,b; Leroux-Roels et al., 2016; Madhi et al., 2016). Pre-clinical studies of a pentavalent vaccine, based on serotypes Ia, Ib, II, III, V are currently under investigation (Kobayashi et al., 2016a). In addition to serotype-specificity, the development of a GBS vaccine with global relevance is a real challenge due to geographic serotype diversity and variations over time. Hence, new vaccine candidates based on conserved surface proteins have been investigated and these included ScpB, Lmb, surface immunogenic protein Sip, leucine rich repeat protein LrrG, Rib and tandem-repeat containing α and β components of the C protein (Wilkinson and Eagon, 1971; Russell-Jones et al., 1984; Michel et al., 1991; Madoff et al., 1992; Stålhammar-Carlemalm et al., 1993; Spellerberg et al., 1999; Cheng et al., 2001; Heath and Feldman, 2005; Seepersaud et al., 2005). A Phase I clinical trial revealed the immunogenicity of Rib and α-C surface proteins against invasive GBS disease (Madhi and Dangor, 2017). Based on reverse vaccinology utilizing genomics, proteomics, transcriptomics and *in silico* technologies, a panel of potential vaccine targets could be discovered (Johri et al., 2006). A similar approach also lead to the identification of GBS pili (Lauer et al., 2005). However, the development of a pilus-based vaccine has been hampered due to antigenic variations of pili in GBS (Margarit et al., 2009; Nuccitelli et al., 2011), but structural vaccinology may overcome this problem. Crystallographic structure of the BP-2a pilus subunit showed four IgG-like domains D1, D2, D3, and D4. Since the D3 domain elicited similar protection as the whole BP-2a component, a promising hybrid vaccine candidate consisting of 6xD3 of the D3 domains from six variant BP-2a pilus subunits was constructed. It is expected to confer protection against all six pilus variants found in GBS strains (Nuccitelli et al., 2011). Other promising vaccine targets that have been identified by reverse vaccinology include the Sip and BibA proteins (Maione et al., 2005; Santi et al., 2007, 2009b). It has been recently shown that a reduced likelihood of maternal GBS acquisition during pregnancy is directly associated with Sip and BibA induced antibodies (Dzanibe et al., 2017). The successful development and introduction of a GBS vaccine may, therefore, result in converting GBS into a predominantly harmless commensal of the mucosa.

## Conclusion

Group B streptococcus adherence and colonization is a complex multifactorial process which determines the success of a pathobiont in the human ecosystems. GBS possess strict regulatory systems which are responsible for fine-tuning the expression of adhesins in order to optimize GBS fitness under the prevailing environmental conditions. Long-term colonization and persistence is largely dependent on the ability of GBS to avoid the host immune response. Over the last two decades, a detailed knowledge about molecular adhesion mechanisms was accumulated for GBS, which considerably enhances our understanding of disease development. These GBS adhesion determinants may represent novel targets for the development of innovative treatment and prophylaxis strategies aiming at control and prevention of invasive GBS disease in neonates as well as adult patients. To date, the sialic acid-containing CPS that underlies the serotyping system of GBS has been the main focus of most vaccine developments. GBS colonizers represent the silent reservoirs responsible for serotype diversity and evolutionary changes in virulence determinants. Investigation of GBS population structure is essential to monitor the circulating serotypes and identify changes in strains causing GBS disease. An aspect, which is especially important in view of a possible serotype-dependent vaccination in the near future.

## Author Contributions

All authors listed have made a substantial, direct and intellectual contribution to the work, and approved it for publication.

## Conflict of Interest Statement

The authors declare that the research was conducted in the absence of any commercial or financial relationships that could be construed as a potential conflict of interest.

## References

[B1] Al Safadi R. Amor S. Hery-Arnaud G. Spellerberg B. Lanotte P. Mereghetti L. (2010). Enhanced expression of *lmb* gene encoding laminin-binding protein in *Streptococcus agalactiae* strains harboring IS*1548* in *scpB-lmb* intergenic region. *PLoS One* 5:e10794. 10.1371/journal.pone.0010794 20520730 PMC2875397

[B2] Al Safadi R. Mereghetti L. Salloum M. Lartigue M. F. Virlogeux-Payant I. Quentin R. (2011). Two-component system RgfA/C activates the *fbsB* gene encoding major fibrinogen-binding protein in highly virulent CC17 clone group B *Streptococcus*. *PLoS One* 6:e14658. 10.1371/journal.pone.0014658 21326613 PMC3033900

[B3] Alhhazmi A. Hurteau D. Tyrrell G. J. (2016). Epidemiology of invasive group B streptococcal disease in Alberta. Canada, from 2003 to 2013. *J. Clin. Microbiol.* 54 1774–1781. 10.1128/jcm.00355-16 27098960 PMC4922093

[B4] Almeida A. Villain A. Joubrel C. Touak G. Sauvage E. Rosinski-Chupin I. (2015). Whole-genome comparison uncovers genomic mutations between group B streptococci sampled from infected newborns and their mothers. *J. Bacteriol.* 197 3354–3366. 10.1128/jb.00429-15 26283765 PMC4573720

[B5] Amin A. Abdulrazzaq Y. M. Uduman S. (2002). Group B streptococcal serotype distribution of isolates from colonized pregnant women at the time of delivery in United Arab Emirates. *J. Infect.* 45 42–46. 10.1053/jinf.2001.0990 12217731

[B6] Anthony B. F. Okada D. M. Hobel C. J. (1979). Epidemiology of the group B streptococcus: maternal and nosocomial sources for infant acquisitions. *J. Pediatr.* 95 431–436. 10.1016/S0022-3476(79)80530-2 381619

[B7] Baker C. J. Carey V. J. Rench M. A. Edwards M. S. Hillier S. L. Kasper D. L. (2014). Maternal antibody at delivery protects neonates from early onset group B streptococcal disease. *J. Infect. Dis.* 209 781–788. 10.1093/infdis/jit549 24133184 PMC3923540

[B8] Baker C. J. Kasper D. L. (1976). Correlation of maternal antibody deficiency with susceptibility to neonatal group B streptococcal infection. *N. Engl. J. Med.* 294 753–756. 10.1056/nejm197604012941404 768760

[B9] Barcaite E. Bartusevicius A. Tameliene R. Kliucinskas M. Maleckiene L. Nadisauskiene R. (2008). Prevalence of maternal group B streptococcal colonisation in European countries. *Acta Obstet. Gynecol. Scand.* 87 260–271. 10.1080/00016340801908759 18307064

[B10] Bellais S. Six A. Fouet A. Longo M. Dmytruk N. Glaser P. (2012). Capsular switching in group B *Streptococcus* CC17 hypervirulent clone: a future challenge for polysaccharide vaccine development. *J. Infect. Dis.* 206 1745–1752. 10.1093/infdis/jis605 23002446

[B11] Bisharat N. Crook D. W. Leigh J. Harding R. M. Ward P. N. Coffey T. J. (2004). Hyperinvasive neonatal group B streptococcus has arisen from a bovine ancestor. *J. Clin. Microbiol.* 42 2161–2167. 10.1128/JCM.42.5.2161-2167.2004 15131184 PMC404684

[B12] Bohnsack J. F. Widjaja K. Ghazizadeh S. Rubens C. E. Hillyard D. R. Parker C. J. (1997). A role for C5 and C5a-ase in the acute neutrophil response to group B streptococcal infections. *J. Infect. Dis.* 175 847–855. 10.1086/513981 9086140

[B13] Brochet M. Couve E. Zouine M. Vallaeys T. Rusniok C. Lamy M. C. (2006). Genomic diversity and evolution within the species *Streptococcus agalactiae*. *Microbes Infect.* 8 1227–1243. 10.1016/j.micinf.2005.11.010 16529966

[B14] Broker G. Spellerberg B. (2004). Surface proteins of *Streptococcus agalactiae* and horizontal gene transfer. *Int. J. Med. Microbiol.* 294 169–175. 10.1016/j.ijmm.2004.06.018 15493827

[B15] Brueggemann A. B. Pai R. Crook D. W. Beall B. (2007). Vaccine escape recombinants emerge after pneumococcal vaccination in the United States. *PLoS Pathog.* 3:e168. 10.1371/journal.ppat.0030168 18020702 PMC2077903

[B16] Buscetta M. Papasergi S. Firon A. Pietrocola G. Biondo C. Mancuso G. (2014). FbsC, a novel fibrinogen-binding protein, promotes *Streptococcus agalactiae*-host cell interactions. *J. Biol. Chem.* 289 21003–21015. 10.1074/jbc.M114.553073 24904056 PMC4110306

[B17] Chang Y. C. Olson J. Beasley F. C. Tung C. Zhang J. Crocker P. R. (2014a). Group B Streptococcus engages an inhibitory Siglec through sialic acid mimicry to blunt innate immune and inflammatory responses *in vivo*. *PLoS Pathog.* 10:e1003846. 10.1371/journal.ppat.1003846 24391502 PMC3879367

[B18] Chang Y. C. Olson J. Louie A. Crocker P. R. Varki A. Nizet V. (2014b). Role of macrophage sialoadhesin in host defense against the sialylated pathogen group B *Streptococcus*. *J. Mol. Med.* 92 951–959. 10.1007/s00109-014-1157-y 24788876 PMC4133643

[B19] Chattopadhyay D. Carey A. J. Caliot E. Webb R. I. Layton J. R. Wang Y. (2011). Phylogenetic lineage and pilus protein Spb1/SAN1518 affect opsonin-independent phagocytosis and intracellular survival of Group B Streptococcus. *Microbes Infect.* 13 369–382. 10.1016/j.micinf.2010.12.009 21238599 PMC4500112

[B20] Cheng Q. Carlson B. Pillai S. Eby R. Edwards L. Olmsted S. B. (2001). Antibody against surface-bound C5a peptidase is opsonic and initiates macrophage killing of group B streptococci. *Infect. Immun.* 69 2302–2308. 10.1128/iai.69.4.2302-2308.2001 11254587 PMC98159

[B21] Cheng Q. Stafslien D. Purushothaman S. S. Cleary P. (2002). The group B streptococcal C5a peptidase is both a specific protease and an invasin. *Infect. Immun.* 70 2408–2413. 10.1128/IAI.70.5.2408-2413.2002 11953377 PMC127948

[B22] Chmouryguina I. Suvorov A. Ferrieri P. Cleary P. P. (1996). Conservation of the C5a peptidase genes in group A and B streptococci. *Infect. Immun.* 64 2387–2390. 8698456 10.1128/iai.64.7.2387-2390.1996 PMC174087

[B23] Costerton J. W. Stewart P. S. Greenberg E. P. (1999). Bacterial biofilms: a common cause of persistent infections. *Science* 284 1318–1322. 10.1126/science.284.5418.1318 10334980

[B24] Cozzi R. Malito E. Lazzarin M. Nuccitelli A. Castagnetti A. Bottomley M. J. (2015). Structure and assembly of group B streptococcus pilus 2b backbone protein. *PLoS One* 10:e0125875. 10.1371/journal.pone.0125875 25942637 PMC4420484

[B25] Da Cunha V. Davies M. R. Douarre P. E. Rosinski-Chupin I. Margarit I. Spinali S. (2014). *Streptococcus agalactiae* clones infecting humans were selected and fixed through the extensive use of tetracycline. *Nat. Commun.* 5:4544. 10.1038/ncomms5544 25088811 PMC4538795

[B26] Dermer P. Lee C. Eggert J. Few B. (2004). A history of neonatal group B streptococcus with its related morbidity and mortality rates in the United States. *J. Pediatr. Nurs.* 19 357–363. 10.1016/j.pedn.2004.05.012 15614260

[B27] Dhanoa A. Karunakaran R. Puthucheary S. D. (2010). Serotype distribution and antibiotic susceptibility of group B streptococci in pregnant women. *Epidemiol. Infect.* 138 979–981. 10.1017/s0950268809991105 19889253

[B28] Di Palo B. Rippa V. Santi I. Brettoni C. Muzzi A. Metruccio M. M. (2013). Adaptive response of Group B streptococcus to high glucose conditions: new insights on the CovRS regulation network. *PLoS One* 8:e61294. 10.1371/journal.pone.0061294 23585887 PMC3621830

[B29] Diedrick M. J. Flores A. E. Hillier S. L. Creti R. Ferrieri P. (2010). Clonal analysis of colonizing group B Streptococcus, serotype IV, an emerging pathogen in the United States. *J. Clin. Microbiol.* 48 3100–3104. 10.1128/jcm.00277-10 20610684 PMC2937746

[B30] Donders G. G. Halperin S. A. Devlieger R. Baker S. Forte P. Wittke F. (2016). Maternal immunization with an investigational trivalent group B streptococcal vaccine: a randomized controlled trial. *Obstet. Gynecol.* 127 213–221. 10.1097/aog.0000000000001190 26942345

[B31] Dramsi S. Caliot E. Bonne I. Guadagnini S. Prevost M. C. Kojadinovic M. (2006). Assembly and role of pili in group B streptococci. *Mol. Microbiol.* 60 1401–1413. 10.1111/j.1365-2958.2006.05190.x 16796677

[B32] D’Urzo N. Martinelli M. Pezzicoli A. De Cesare V. Pinto V. Margarit I. (2014). Acidic pH strongly enhances *in vitro* biofilm formation by a subset of hypervirulent ST-17 *Streptococcus agalactiae* strains. *Appl. Environ. Microbiol.* 80 2176–2185. 10.1128/aem.03627-13 24487536 PMC3993151

[B33] Dzanibe S. Kwatra G. Adrian P. V. Kimaro-Mlacha S. Z. Cutland C. L. Madhi S. A. (2017). Association between antibodies against group B *Streptococcus* surface proteins and recto-vaginal colonisation during pregnancy. *Sci. Rep.* 7:16454. 10.1038/s41598-017-16757-9 29184151 PMC5705700

[B34] Ekelund K. Slotved H. C. Nielsen H. U. Kaltoft M. S. Konradsen H. B. (2003). Emergence of invasive serotype VIII group B streptococcal infections in Denmark. *J. Clin. Microbiol.* 41 4442–4444. 10.1128/JCM.41.9.4442-4444.2003 12958288 PMC193804

[B35] Fabbrini M. Rigat F. Rinaudo C. D. Passalaqua I. Khacheh S. Creti R. (2016). The protective value of maternal group B *Streptococcus* antibodies: quantitative and functional analysis of naturally acquired responses to capsular polysaccharides and pilus proteins in European maternal sera. *Clin. Infect. Dis.* 63 746–753. 10.1093/cid/ciw377 27402816

[B36] Faralla C. Metruccio M. M. De Chiara M. Mu R. Patras K. A. Muzzi A. (2014). Analysis of two-component systems in group B Streptococcus shows that RgfAC and the novel FspSR modulate virulence and bacterial fitness. *mBio* 5:e870-14. 10.1128/mBio.00870-14 24846378 PMC4030450

[B37] Farley M. M. (2001). Group B streptococcal disease in nonpregnant adults. *Clin. Infect. Dis.* 33 556–561. 10.1086/322696 11462195

[B38] Ferrieri P. Lynfield R. Creti R. Flores A. E. (2013). Serotype IV and invasive group B Streptococcus disease in neonates. Minnesota, USA, 2000-2010. *Emerg. Infect. Dis.* 19 551–558. 10.3201/eid1904.121572 23628320 PMC3647718

[B39] Flores A. R. Galloway-Pena J. Sahasrabhojane P. Saldana M. Yao H. Su X. (2015). Sequence type 1 group B Streptococcus, an emerging cause of invasive disease in adults, evolves by small genetic changes. *Proc. Natl. Acad. Sci. U.S.A.* 112 6431–6436. 10.1073/pnas.1504725112 25941374 PMC4443349

[B40] Florindo C. Damiao V. Silvestre I. Farinha C. Rodrigues F. Nogueira F. (2014). Epidemiological surveillance of colonising group B Streptococcus epidemiology in the Lisbon and Tagus Valley regions, Portugal (2005 to 2012): emergence of a new epidemic type IV/clonal complex 17 clone. *Euro. Surveill.* 19:20825. 10.2807/1560-7917.ES2014.19.23.20825 24957747

[B41] Franken C. Haase G. Brandt C. Weber-Heynemann J. Martin S. Lammler C. (2001). Horizontal gene transfer and host specificity of beta-haemolytic streptococci: the role of a putative composite transposon containing scpB and lmb. *Mol. Microbiol.* 41 925–935. 10.1046/j.1365-2958.2001.02563.x 11532154

[B42] Fry R. M. (1938). Fatal infections by hemolytic streptococcus group B. *Lancet* 231 199–201. 10.1016/S0140-6736(00)93202-1

[B43] Gherardi G. Imperi M. Baldassarri L. Pataracchia M. Alfarone G. Recchia S. (2007). Molecular epidemiology and distribution of serotypes, surface proteins, and antibiotic resistance among group B streptococci in Italy. *J. Clin. Microbiol.* 45 2909–2916. 10.1128/jcm.00999-07 17634303 PMC2045288

[B44] Granlund M. Michel F. Norgren M. (2001). Mutually exclusive distribution of IS*1548* and GBSi1, an active group II intron identified in human isolates of group B streptococci. *J. Bacteriol.* 183 2560–2569. 10.1128/jb.183.8.2560-2569.2001 11274116 PMC95173

[B45] Gutekunst H. Eikmanns B. J. Reinscheid D. J. (2004). The novel fibrinogen-binding protein FbsB promotes *Streptococcus agalactiae* invasion into epithelial cells. *Infect. Immun.* 72 3495–3504. 10.1128/iai.72.6.3495-3504.2004 15155657 PMC415667

[B46] Hansen S. M. Uldbjerg N. Kilian M. Sorensen U. B. (2004). Dynamics of *Streptococcus agalactiae* colonization in women during and after pregnancy and in their infants. *J. Clin. Microbiol.* 42 83–89. 10.1128/JCM.42.1.83-89.2004 14715736 PMC321715

[B47] Heath P. T. Culley F. J. Jones C. E. Kampmann B. Le Doare K. Nunes M. C. (2017). Group B streptococcus and respiratory syncytial virus immunisation during pregnancy: a landscape analysis. *Lancet Infect. Dis.* 17 e223–e234. 10.1016/s1473-3099(17)30232-3 28433702

[B48] Heath P. T. Feldman R. G. (2005). Vaccination against group B streptococcus. *Expert Rev. Vaccines* 4 207–218. 10.1586/14760584.4.2.207 15889994

[B49] Héry-Arnaud G. Bruant G. Lanotte P. Brun S. Picard B. Rosenau A. (2007). Mobile genetic elements provide evidence for a bovine origin of clonal complex 17 of *Streptococcus agalactiae*. *Appl. Environ. Microbiol.* 73 4668–4672. 10.1128/aem.02604-06 17526784 PMC1932819

[B50] Heyderman R. S. Madhi S. A. French N. Cutland C. Ngwira B. Kayambo D. (2016). Group B streptococcus vaccination in pregnant women with or without HIV in Africa: a non-randomised phase 2, open-label, multicentre trial. *Lancet Infect. Dis.* 16 546–555. 10.1016/s1473-3099(15)00484-3 26869376 PMC4835545

[B51] Hickman M. E. Rench M. A. Ferrieri P. Baker C. J. (1999). Changing epidemiology of group B streptococcal colonization. *Pediatrics* 104 203–209. 10.1542/peds.104.2.203 10428995

[B52] Ho Y. R. Li C. M. Yu C. H. Lin Y. J. Wu C. M. Harn I. C. (2013). The enhancement of biofilm formation in Group B streptococcal isolates at vaginal pH. *Med. Microbiol. Immunol.* 202 105–115. 10.1007/s00430-012-0255-0 22797522

[B53] Hood M. Janney A. Dameron G. (1961). Beta hemolytic streptococcus group B associated with problems of the perinatal period. *Am. J. Obstet. Gynecol.* 82 809–818. 10.1016/S0002-9378(16)36146-4 13908742

[B54] Hoshina K. Suzuki Y. Nishida H. Kaneko K. Matsuda S. Kobayashi M. (2002). Trend of neonatal group B streptococcal infection during the last 15 years. *Pediatr. Int.* 44 641–646. 10.1046/j.1442-200X.2002.01638.x 12421262

[B55] Ippolito D. L. James W. A. Tinnemore D. Huang R. R. Dehart M. J. Williams J. (2010). Group B streptococcus serotype prevalence in reproductive-age women at a tertiary care military medical center relative to global serotype distribution. *BMC Infect. Dis.* 10:336. 10.1186/1471-2334-10-336 21106080 PMC3004907

[B56] Jiang S. Park S. E. Yadav P. Paoletti L. C. Wessels M. R. (2012). Regulation and function of pilus island 1 in group B streptococcus. *J. Bacteriol.* 194 2479–2490. 10.1128/jb.00202-12 22408160 PMC3347183

[B57] Jiang S. Wessels M. R. (2014). BsaB, a novel adherence factor of group B *Streptococcus*. *Infect. Immun.* 82 1007–1016. 10.1128/iai.01014-13 24343649 PMC3957996

[B58] Jiang S. M. Cieslewicz M. J. Kasper D. L. Wessels M. R. (2005). Regulation of virulence by a two-component system in group B streptococcus. *J. Bacteriol.* 187 1105–1113. 10.1128/jb.187.3.1105-1113.2005 15659687 PMC545708

[B59] Jiang S. M. Ishmael N. Dunning Hotopp J. Puliti M. Tissi L. Kumar N. (2008). Variation in the group B *Streptococcus* CsrRS regulon and effects on pathogenicity. *J. Bacteriol.* 190 1956–1965. 10.1128/jb.01677-07 18203834 PMC2258897

[B60] Johri A. K. Paoletti L. C. Glaser P. Dua M. Sharma P. K. Grandi G. (2006). Group B *Streptococcus*: global incidence and vaccine development. *Nat. Rev. Microbiol.* 4 932–942. 10.1038/nrmicro1552 17088932 PMC2742968

[B61] Jones N. Bohnsack J. F. Takahashi S. Oliver K. A. Chan M. S. Kunst F. (2003). Multilocus sequence typing system for group B streptococcus. *J. Clin. Microbiol.* 41 2530–2536. 10.1128/JCM.41.6.2530-2536.2003 12791877 PMC156480

[B62] Karunakaran R. Raja N. S. Hafeez A. Puthucheary S. D. (2009). Group B Streptococcus infection: epidemiology, serotypes, and antimicrobial susceptibility of selected isolates in the population beyond infancy (excluding females with genital tract- and pregnancy-related isolates) at the University Malaya Medical Centre, Kuala Lumpur. *Jpn. J. Infect. Dis.* 62 192–194. 19468178

[B63] Keefe G. P. (1997). *Streptococcus agalactiae* mastitis: a review. *Can. Vet. J.* 38 429–437. 9220132 PMC1576741

[B64] Kobayashi M. Schrag S. J. Alderson M. R. Madhi S. A. Baker C. J. Sobanjo-Ter Meulen A. (2016a). WHO consultation on group B *Streptococcus* vaccine development: report from a meeting held on 27-28 April 2016. *Vaccine.* 10.1016/j.vaccine.2016.12.029 [Epub ahead of print]. 28017431 PMC6892266

[B65] Kobayashi M. Vekemans J. Baker C. J. Ratner A. J. Le Doare K. Schrag S. J. (2016b). Group B *Streptococcus* vaccine development: present status and future considerations, with emphasis on perspectives for low and middle income countries. *F1000Res* 5:2355. 10.12688/f1000research.9363.1 27803803 PMC5070600

[B66] Konto-Ghiorghi Y. Mairey E. Mallet A. Dumenil G. Caliot E. Trieu-Cuot P. (2009). Dual role for pilus in adherence to epithelial cells and biofilm formation in *Streptococcus agalactiae*. *PLoS Pathog.* 5:e1000422. 10.1371/journal.ppat.1000422 19424490 PMC2674936

[B67] Krishnan V. Gaspar A. H. Ye N. Mandlik A. Ton-That H. Narayana S. V. (2007). An IgG-like domain in the minor pilin GBS52 of *Streptococcus agalactiae* mediates lung epithelial cell adhesion. *Structure* 15 893–903. 10.1016/j.str.2007.06.015 17697995 PMC2844079

[B68] Lachenauer C. S. Kasper D. L. Shimada J. Ichiman Y. Ohtsuka H. Kaku M. (1999). Serotypes VI and VIII predominate among group B streptococci isolated from pregnant Japanese women. *J. Infect. Dis.* 179 1030–1033. 10.1086/314666 10068604

[B69] Lamagni T. L. Keshishian C. Efstratiou A. Guy R. Henderson K. L. Broughton K. (2013). Emerging trends in the epidemiology of invasive group B streptococcal disease in England and Wales, 1991-2010. *Clin. Infect. Dis.* 57 682–688. 10.1093/cid/cit337 23845950

[B70] Lamy M. C. Dramsi S. Billoet A. Reglier-Poupet H. Tazi A. Raymond J. (2006). Rapid detection of the “highly virulent” group B Streptococcus ST-17 clone. *Microbes Infect.* 8 1714–1722. 10.1016/j.micinf.2006.02.008 16822689

[B71] Lamy M. C. Zouine M. Fert J. Vergassola M. Couve E. Pellegrini E. (2004). CovS/CovR of group B streptococcus: a two-component global regulatory system involved in virulence. *Mol. Microbiol.* 54 1250–1268. 10.1111/j.1365-2958.2004.04365.x 15554966

[B72] Lancefield R. C. (1938). Two Serological Types Of Group B hemolytic streptococci with related. But not identical, type-specific substances. *J. Exp. Med.* 67 25–40. 10.1084/jem.67.1.25 19870707 PMC2133550

[B73] Landwehr-Kenzel S. Henneke P. (2014). Interaction of *Streptococcus agalactiae* and cellular innate immunity in colonization and disease. *Front. Immunol.* 5:519. 10.3389/fimmu.2014.00519 25400631 PMC4212683

[B74] Lartigue M. F. Hery-Arnaud G. Haguenoer E. Domelier A. S. Schmit P. O. van der Mee-Marquet N. (2009). Identification of *Streptococcus agalactiae* isolates from various phylogenetic lineages by matrix-assisted laser desorption ionization-time of flight mass spectrometry. *J. Clin. Microbiol.* 47 2284–2287. 10.1128/jcm.00175-09 19403759 PMC2708490

[B75] Lauer P. Rinaudo C. D. Soriani M. Margarit I. Maione D. Rosini R. (2005). Genome analysis reveals pili in Group B *Streptococcus*. *Science* 309:105. 10.1126/science.1111563 15994549

[B76] Lazzarin M. Mu R. Fabbrini M. Ghezzo C. Rinaudo C. D. Doran K. S. (2017). Contribution of pilus type 2b to invasive disease caused by a *Streptococcus agalactiae* ST-17 strain. *BMC Microbiol.* 17:148. 10.1186/s12866-017-1057-8 28673237 PMC5496222

[B77] Le Doare K. Heath P. T. (2013). An overview of global GBS epidemiology. *Vaccine* 31(Suppl. 4), D7–D12. 10.1016/j.vaccine.2013.01.009 23973349

[B78] Le Doare K. Kampmann B. (2014). Breast milk and Group B streptococcal infection: vector of transmission or vehicle for protection? *Vaccine* 32 3128–3132. 10.1016/j.vaccine.2014.04.020 24736004 PMC4037808

[B79] Lemire P. Houde M. Lecours M. P. Fittipaldi N. Segura M. (2012). Role of capsular polysaccharide in Group B Streptococccus interactions with dendritic cells. *Microbes Infect.* 14 1064–1076. 10.1016/j.micinf.2012.05.015 22683668

[B80] Leroux-Roels G. Maes C. Willekens J. De Boever F. de Rooij R. Martell L. (2016). A randomized, observer-blind Phase Ib study to identify formulations and vaccine schedules of a trivalent Group B Streptococcus vaccine for use in non-pregnant and pregnant women. *Vaccine* 34 1786–1791. 10.1016/j.vaccine.2016.02.044 26928074

[B81] Lewis K. (2005). Persister cells and the riddle of biofilm survival. *Biochemistry* 70 267–274. 10.1007/s10541-005-0111-6 15807669

[B82] Libster R. Edwards K. M. Levent F. Edwards M. S. Rench M. A. Castagnini L. A. (2012). Long-term outcomes of group B streptococcal meningitis. *Pediatrics* 130 e8–e15. 10.1542/peds.2011-3453 22689869

[B83] Lin F. Y. Philips J. B. III Azimi P. H. Weisman L. E. Clark P. Rhoads G. G. (2001). Level of maternal antibody required to protect neonates against early-onset disease caused by group B Streptococcus type Ia: a multicenter, seroepidemiology study. *J. Infect. Dis.* 184 1022–1028. 10.1086/323350 11574917

[B84] Lin F. Y. Weisman L. E. Azimi P. H. Philips J. B. III Clark P. Regan J. (2004). Level of maternal IgG anti-group B streptococcus type III antibody correlated with protection of neonates against early-onset disease caused by this pathogen. *J. Infect. Dis.* 190 928–934. 10.1086/422756 15295698

[B85] Lin H. C. Chen C. J. Chiang K. H. Yen T. Y. Ho C. M. Hwang K. P. (2016). Clonal dissemination of invasive and colonizing clonal complex 1 of serotype VI group B *Streptococcus* in central Taiwan. *J. Microbiol. Immunol. Infect.* 49 902–909. 10.1016/j.jmii.2014.11.002 25560254

[B86] Lindahl G. Stalhammar-Carlemalm M. Areschoug T. (2005). Surface proteins of *Streptococcus agalactiae* and related proteins in other bacterial pathogens. *Clin. Microbiol. Rev.* 18 102–127. 10.1128/cmr.18.1.102-127.2005 15653821 PMC544178

[B87] Luan S. L. Granlund M. Norgren M. (2003). An inserted DNA fragment with plasmid features is uniquely associated with the presence of the GBSi1 group II intron in *Streptococcus agalactiae*. *Gene* 312 305–312. 10.1016/S0378-1119(03)00634-6 12909368

[B88] Luan S. L. Granlund M. Sellin M. Lagergard T. Spratt B. G. Norgren M. (2005). Multilocus sequence typing of Swedish invasive group B streptococcus isolates indicates a neonatally associated genetic lineage and capsule switching. *J. Clin. Microbiol.* 43 3727–3733. 10.1128/jcm.43.8.3727-3733.2005 16081902 PMC1233917

[B89] Madhi S. A. Cutland C. L. Jose L. Koen A. Govender N. Wittke F. (2016). Safety and immunogenicity of an investigational maternal trivalent group B streptococcus vaccine in healthy women and their infants: a randomised phase 1b/2 trial. *Lancet Infect. Dis.* 16 923–934. 10.1016/s1473-3099(16)00152-3 27139805

[B90] Madhi S. A. Dangor Z. (2017). Prospects for preventing infant invasive GBS disease through maternal vaccination. *Vaccine* 35 4457–4460. 10.1016/j.vaccine.2017.02.025 28237500

[B91] Madhi S. A. Dangor Z. Heath P. T. Schrag S. Izu A. Sobanjo-Ter Meulen A. (2013). Considerations for a phase-III trial to evaluate a group B Streptococcus polysaccharide-protein conjugate vaccine in pregnant women for the prevention of early- and late-onset invasive disease in young-infants. *Vaccine* 31(Suppl. 4), D52–D57. 10.1016/j.vaccine.2013.02.029 23973347

[B92] Madoff L. C. Michel J. L. Gong E. W. Rodewald A. K. Kasper D. L. (1992). Protection of neonatal mice from group B streptococcal infection by maternal immunization with beta C protein. *Infect. Immun.* 60 4989–4994. 1452329 10.1128/iai.60.12.4989-4994.1992 PMC258267

[B93] Maione D. Margarit I. Rinaudo C. D. Masignani V. Mora M. Scarselli M. (2005). Identification of a universal Group B streptococcus vaccine by multiple genome screen. *Science* 309 148–150. 10.1126/science.1109869 15994562 PMC1351092

[B94] Maisey H. C. Doran K. S. Nizet V. (2008a). Recent advances in understanding the molecular basis of group B streptococcus virulence. *Expert Rev. Mol. Med.* 10:e27. 10.1017/s1462399408000811 18803886 PMC2676346

[B95] Maisey H. C. Hensler M. Nizet V. Doran K. S. (2007). Group B streptococcal pilus proteins contribute to adherence to and invasion of brain microvascular endothelial cells. *J. Bacteriol.* 189 1464–1467. 10.1128/jb.01153-06 17041051 PMC1797338

[B96] Maisey H. C. Quach D. Hensler M. E. Liu G. Y. Gallo R. L. Nizet V. (2008b). A group B streptococcal pilus protein promotes phagocyte resistance and systemic virulence. *FASEB J.* 22 1715–1724. 10.1096/fj.07-093963 18198218 PMC2721339

[B97] Manning S. D. Lewis M. A. Springman A. C. Lehotzky E. Whittam T. S. Davies H. D. (2008). Genotypic diversity and serotype distribution of group B streptococcus isolated from women before and after delivery. *Clin. Infect. Dis.* 46 1829–1837. 10.1086/588296 18462173 PMC9491394

[B98] Manning S. D. Springman A. C. Lehotzky E. Lewis M. A. Whittam T. S. Davies H. D. (2009). Multilocus sequence types associated with neonatal group B streptococcal sepsis and meningitis in Canada. *J. Clin. Microbiol.* 47 1143–1148. 10.1128/jcm.01424-08 19158264 PMC2668308

[B99] Margarit I. Rinaudo C. D. Galeotti C. L. Maione D. Ghezzo C. Buttazzoni E. (2009). Preventing bacterial infections with pilus-based vaccines: the group B streptococcus paradigm. *J. Infect. Dis.* 199 108–115. 10.1086/595564 19086816

[B100] Martins E. R. Pessanha M. A. Ramirez M. Melo-Cristino J. (2007). Analysis of group B streptococcal isolates from infants and pregnant women in Portugal revealing two lineages with enhanced invasiveness. *J. Clin. Microbiol.* 45 3224–3229. 10.1128/jcm.01182-07 17699641 PMC2045366

[B101] Matsubara K. Katayama K. Baba K. Nigami H. Harigaya H. Sugiyama M. (2002). Seroepidemiologic studies of serotype VIII group B Streptococcus in Japan. *J. Infect. Dis.* 186 855–858. 10.1086/342411 12198624

[B102] Melin P. (2011). Neonatal group B streptococcal disease: from pathogenesis to preventive strategies. *Clin. Microbiol. Infect.* 17 1294–1303. 10.1111/j.1469-0691.2011.03576.x 21672083

[B103] Melin P. Efstratiou A. (2013). Group B streptococcal epidemiology and vaccine needs in developed countries. *Vaccine* 31(Suppl. 4), D31–D42. 10.1016/j.vaccine.2013.05.012 23973345

[B104] Meyn L. A. Krohn M. A. Hillier S. L. (2009). Rectal colonization by group B Streptococcus as a predictor of vaginal colonization. *Am. J. Obstet. Gynecol.* 201 76.e1–76.e7. 10.1016/j.ajog.2009.02.011 19371857 PMC2770838

[B105] Michel J. L. Madoff L. C. Kling D. E. Kasper D. L. Ausubel F. M. (1991). Cloned alpha and beta C-protein antigens of group B streptococci elicit protective immunity. *Infect. Immun.* 59 2023–2028. 1674738 10.1128/iai.59.6.2023-2028.1991 PMC257960

[B106] Mu R. Kim B. J. Paco C. Del Rosario Y. Courtney H. S. Doran K. S. (2014). Identification of a group B streptococcal fibronectin binding protein, SfbA, that contributes to invasion of brain endothelium and development of meningitis. *Infect. Immun.* 82 2276–2286. 10.1128/iai.01559-13 24643538 PMC4019170

[B107] Nobbs A. H. Lamont R. J. Jenkinson H. F. (2009). *Streptococcus* adherence and colonization. *Microbiol. Mol. Biol. Rev.* 73 407–450. 10.1128/mmbr.00014-09 19721085 PMC2738137

[B108] Nuccitelli A. Cozzi R. Gourlay L. J. Donnarumma D. Necchi F. Norais N. (2011). Structure-based approach to rationally design a chimeric protein for an effective vaccine against Group B *Streptococcus* infections. *Proc. Natl. Acad. Sci. U.S.A.* 108 10278–10283. 10.1073/pnas.1106590108 21593422 PMC3121859

[B109] Paoletti L. C. Kasper D. L. (2003). Glycoconjugate vaccines to prevent group B streptococcal infections. *Expert Opin. Biol. Ther.* 3 975–984. 10.1517/14712598.3.6.975 12943456

[B110] Paoletti L. J. Bradford J. Paoletti L. C. (1999). A serotype VIII strain among colonizing group B streptococcal isolates in Boston, Massachusetts. *J. Clin. Microbiol.* 37 3759–3760. 10523595 10.1128/jcm.37.11.3759-3760.1999 PMC85754

[B111] Park S. E. Jiang S. Wessels M. R. (2012). CsrRS and environmental pH regulate group B streptococcus adherence to human epithelial cells and extracellular matrix. *Infect. Immun.* 80 3975–3984. 10.1128/iai.00699-12 22949550 PMC3486057

[B112] Parker R. E. Laut C. Gaddy J. A. Zadoks R. N. Davies H. D. Manning S. D. (2016). Association between genotypic diversity and biofilm production in group B Streptococcus. *BMC Microbiol.* 16:86. 10.1186/s12866-016-0704-9 27206613 PMC4875601

[B113] Patras K. A. Wang N. Y. Fletcher E. M. Cavaco C. K. Jimenez A. Garg M. (2013). Group B Streptococcus CovR regulation modulates host immune signalling pathways to promote vaginal colonization. *Cell. Microbiol.* 15 1154–1167. 10.1111/cmi.12105 23298320 PMC3657335

[B114] Pezzicoli A. Ruggiero P. Amerighi F. Telford J. L. Soriani M. (2012). Exogenous sialic acid transport contributes to group B streptococcus infection of mucosal surfaces. *J. Infect. Dis.* 206 924–931. 10.1093/infdis/jis451 22829646

[B115] Pezzicoli A. Santi I. Lauer P. Rosini R. Rinaudo D. Grandi G. (2008). Pilus backbone contributes to group B *Streptococcus* paracellular translocation through epithelial cells. *J. Infect. Dis.* 198 890–898. 10.1086/591182 18694342

[B116] Phares C. R. Lynfield R. Farley M. M. Mohle-Boetani J. Harrison L. H. Petit S. (2008). Epidemiology of invasive group B streptococcal disease in the United States, 1999-2005. *JAMA* 299 2056–2065. 10.1001/jama.299.17.2056 18460666

[B117] Rajagopal L. (2009). Understanding the regulation of group B streptococcal virulence factors. *Future Microbiol.* 4 201–221. 10.2217/17460913.4.2.201 19257847 PMC2691590

[B118] Rato M. G. Bexiga R. Florindo C. Cavaco L. M. Vilela C. L. Santos-Sanches I. (2013). Antimicrobial resistance and molecular epidemiology of streptococci from bovine mastitis. *Vet. Microbiol.* 161 286–294. 10.1016/j.vetmic.2012.07.043 22964008

[B119] Rinaudo C. D. Rosini R. Galeotti C. L. Berti F. Necchi F. Reguzzi V. (2010). Specific involvement of pilus type 2a in biofilm formation in group B Streptococcus. *PLoS One* 5:e9216. 10.1371/journal.pone.0009216 20169161 PMC2821406

[B120] Rosenau A. Martins K. Amor S. Gannier F. Lanotte P. van der Mee-Marquet N. (2007). Evaluation of the ability of *Streptococcus agalactiae* strains isolated from genital and neonatal specimens to bind to human fibrinogen and correlation with characteristics of the fbsA and fbsB genes. *Infect. Immun.* 75 1310–1317. 10.1128/iai.00996-06 17158903 PMC1828567

[B121] Rosini R. Margarit I. (2015). Biofilm formation by *Streptococcus agalactiae*: influence of environmental conditions and implicated virulence factors. *Front. Cell. Infect. Microbiol.* 5:6. 10.3389/fcimb.2015.00006 25699242 PMC4316791

[B122] Rosini R. Rinaudo C. D. Soriani M. Lauer P. Mora M. Maione D. (2006). Identification of novel genomic islands coding for antigenic pilus-like structures in *Streptococcus agalactiae*. *Mol. Microbiol.* 61 126–141. 10.1111/j.1365-2958.2006.05225.x 16824100

[B123] Rubens C. E. Wessels M. R. Heggen L. M. Kasper D. L. (1987). Transposon mutagenesis of type III group B Streptococcus: correlation of capsule expression with virulence. *Proc. Natl. Acad. Sci. U.S.A.* 84 7208–7212. 10.1073/pnas.84.20.7208 2823254 PMC299259

[B124] Russell-Jones G. J. Gotschlich E. C. Blake M. S. (1984). A surface receptor specific for human IgA on group B streptococci possessing the Ibc protein antigen. *J. Exp. Med.* 160 1467–1475. 10.1084/jem.160.5.1467 6387034 PMC2187502

[B125] Salloum M. van der Mee-Marquet N. Valentin-Domelier A. S. Quentin R. (2011). Diversity of prophage DNA regions of *Streptococcus agalactiae* clonal lineages from adults and neonates with invasive infectious disease. *PLoS One* 6:e20256. 10.1371/journal.pone.0020256 21633509 PMC3102099

[B126] Santi I. Grifantini R. Jiang S. M. Brettoni C. Grandi G. Wessels M. R. (2009a). CsrRS regulates group B Streptococcus virulence gene expression in response to environmental pH: a new perspective on vaccine development. *J. Bacteriol.* 191 5387–5397. 10.1128/jb.00370-09 19542277 PMC2725634

[B127] Santi I. Maione D. Galeotti C. L. Grandi G. Telford J. L. Soriani M. (2009b). BibA induces opsonizing antibodies conferring in vivo protection against group B *Streptococcus*. *J. Infect. Dis.* 200 564–570. 10.1086/603540 19586417

[B128] Santi I. Scarselli M. Mariani M. Pezzicoli A. Masignani V. Taddei A. (2007). BibA: a novel immunogenic bacterial adhesin contributing to group B *Streptococcus* survival in human blood. *Mol. Microbiol.* 63 754–767. 10.1111/j.1365-2958.2006.05555.x 17212592

[B129] Schrag S. J. Verani J. R. (2013). Intrapartum antibiotic prophylaxis for the prevention of perinatal group B streptococcal disease: experience in the United States and implications for a potential group B streptococcal vaccine. *Vaccine* 31(Suppl. 4), D20–D26. 10.1016/j.vaccine.2012.11.056 23219695 PMC11843781

[B130] Schubert A. Zakikhany K. Pietrocola G. Meinke A. Speziale P. Eikmanns B. J. (2004). The fibrinogen receptor FbsA promotes adherence of *Streptococcus agalactiae* to human epithelial cells. *Infect. Immun.* 72 6197–6205. 10.1128/iai.72.11.6197-6205.2004 15501744 PMC523014

[B131] Schuchat A. (1998). Epidemiology of group B streptococcal disease in the United States: shifting paradigms. *Clin. Microbiol. Rev.* 11 497–513. 9665980 10.1128/cmr.11.3.497 PMC88893

[B132] Seepersaud R. Hanniffy S. B. Mayne P. Sizer P. Le Page R. Wells J. M. (2005). Characterization of a novel leucine-rich repeat protein antigen from group B streptococci that elicits protective immunity. *Infect. Immun.* 73 1671–1683. 10.1128/iai.73.3.1671-1683.2005 15731068 PMC1064916

[B133] Seifert K. N. Adderson E. E. Whiting A. A. Bohnsack J. F. Crowley P. J. Brady L. J. (2006). A unique serine-rich repeat protein (Srr-2) and novel surface antigen (epsilon) associated with a virulent lineage of serotype III *Streptococcus agalactiae*. *Microbiology* 152 1029–1040. 10.1099/mic.0.28516-0 16549667

[B134] Sendi P. Johansson L. Norrby-Teglund A. (2008). Invasive group B Streptococcal disease in non-pregnant adults : a review with emphasis on skin and soft-tissue infections. *Infection* 36 100–111. 10.1007/s15010-007-7251-0 18193384

[B135] Seo H. S. Minasov G. Seepersaud R. Doran K. S. Dubrovska I. Shuvalova L. (2013). Characterization of fibrinogen binding by glycoproteins Srr1 and Srr2 of *Streptococcus agalactiae*. *J. Biol. Chem.* 288 35982–35996. 10.1074/jbc.M113.513358 24165132 PMC3861647

[B136] Seo H. S. Mu R. Kim B. J. Doran K. S. Sullam P. M. (2012). Binding of glycoprotein Srr1 of *Streptococcus agalactiae* to fibrinogen promotes attachment to brain endothelium and the development of meningitis. *PLoS Pathog.* 8:e1002947. 10.1371/journal.ppat.1002947 23055927 PMC3464228

[B137] Shabayek S. Abdalla S. Abouzeid A. M. (2014). Serotype and surface protein gene distribution of colonizing group B streptococcus in women in Egypt. *Epidemiol. Infect.* 142 208–210. 10.1017/s0950268813000848 23561305 PMC9152610

[B138] Sheen T. R. Jimenez A. Wang N. Y. Banerjee A. van Sorge N. M. Doran K. S. (2011). Serine-rich repeat proteins and pili promote *Streptococcus agalactiae* colonization of the vaginal tract. *J. Bacteriol.* 193 6834–6842. 10.1128/jb.00094-11 21984789 PMC3232834

[B139] Singh B. Fleury C. Jalalvand F. Riesbeck K. (2012). Human pathogens utilize host extracellular matrix proteins laminin and collagen for adhesion and invasion of the host. *FEMS Microbiol. Rev.* 36 1122–1180. 10.1111/j.1574-6976.2012.00340.x 22537156

[B140] Skoff T. H. Farley M. M. Petit S. Craig A. S. Schaffner W. Gershman K. (2009). Increasing burden of invasive group B streptococcal disease in nonpregnant adults, 1990-2007. *Clin. Infect. Dis.* 49 85–92. 10.1086/599369 19480572

[B141] Slotved H. C. Kong F. Lambertsen L. Sauer S. Gilbert G. L. (2007). Serotype IX, a proposed new *Streptococcus agalactiae* serotype. *J. Clin. Microbiol.* 45 2929–2936. 10.1128/jcm.00117-07 17634306 PMC2045254

[B142] Sørensen U. B. S. Poulsen K. Ghezzo C. Margarit I. Kilian M. (2010). Emergence and global dissemination of host-specific *Streptococcus agalactiae* clones. *mBio* 1:e00178-10. 10.1128/mBio.00178-10 20824105 PMC2932510

[B143] Spellerberg B. Rozdzinski E. Martin S. Weber-Heynemann J. Schnitzler N. Lutticken R. (1999). Lmb, a protein with similarities to the LraI adhesin family, mediates attachment of *Streptococcus agalactiae* to human laminin. *Infect. Immun.* 67 871–878. 9916102 10.1128/iai.67.2.871-878.1999 PMC96398

[B144] Springman A. C. Lacher D. W. Waymire E. A. Wengert S. L. Singh P. Zadoks R. N. (2014). Pilus distribution among lineages of group b streptococcus: an evolutionary and clinical perspective. *BMC Microbiol.* 14:159. 10.1186/1471-2180-14-159 24943359 PMC4074840

[B145] Stålhammar-Carlemalm M. Stenberg L. Lindahl G. (1993). Protein rib: a novel group B streptococcal cell surface protein that confers protective immunity and is expressed by most strains causing invasive infections. *J. Exp. Med.* 177 1593–1603. 10.1084/jem.177.6.1593 8496678 PMC2191029

[B146] Stoner T. D. Weston T. A. Trejo J. Doran K. S. (2015). Group B streptococcal infection and activation of human astrocytes. *PLoS One* 10:e0128431. 10.1371/journal.pone.0128431 26030618 PMC4452173

[B147] Sukhnanand S. Dogan B. Ayodele M. O. Zadoks R. N. Craver M. P. Dumas N. B. (2005). Molecular subtyping and characterization of bovine and human *Streptococcus agalactiae* isolates. *J. Clin. Microbiol.* 43 1177–1186. 10.1128/jcm.43.3.1177-1186.2005 15750080 PMC1081236

[B148] Tamura G. S. Kuypers J. M. Smith S. Raff H. Rubens C. E. (1994). Adherence of group B streptococci to cultured epithelial cells: roles of environmental factors and bacterial surface components. *Infect. Immun.* 62 2450–2458. 8188370 10.1128/iai.62.6.2450-2458.1994 PMC186531

[B149] Tazi A. Disson O. Bellais S. Bouaboud A. Dmytruk N. Dramsi S. (2010). The surface protein HvgA mediates group B streptococcus hypervirulence and meningeal tropism in neonates. *J. Exp. Med.* 207 2313–2322. 10.1084/jem.20092594 20956545 PMC2964583

[B150] Teatero S. Athey T. B. Van Caeseele P. Horsman G. Alexander D. C. Melano R. G. (2015). Emergence of serotype IV group B *Streptococcus* adult invasive disease in Manitoba and Saskatchewan. Canada, is driven by clonal sequence type 459 strains. *J. Clin. Microbiol.* 53 2919–2926. 10.1128/jcm.01128-15 26135871 PMC4540936

[B151] Teatero S. Ferrieri P. Fittipaldi N. (2016). Serotype IV sequence type 468 group B *Streptococcus* neonatal invasive disease, Minnesota, USA. *Emerg. Infect. Dis.* 22 1937–1940. 10.3201/eid2211.152031 27767922 PMC5088005

[B152] Teatero S. Ferrieri P. Martin I. Demczuk W. McGeer A. Fittipaldi N. (2017). Serotype distribution, population structure, and antimicrobial resistance of group B *Streptococcus* strains recovered from colonized pregnant women. *J. Clin. Microbiol.* 55 412–422. 10.1128/jcm.01615-16 27852675 PMC5277510

[B153] Teatero S. McGeer A. Low D. E. Li A. Demczuk W. Martin I. (2014). Characterization of invasive group B streptococcus strains from the greater Toronto area, Canada. *J. Clin. Microbiol.* 52 1441–1447. 10.1128/jcm.03554-13 24554752 PMC3993709

[B154] Tenenbaum T. Spellerberg B. Adam R. Vogel M. Kim K. S. Schroten H. (2007). *Streptococcus agalactiae* invasion of human brain microvascular endothelial cells is promoted by the laminin-binding protein Lmb. *Microbes Infect.* 9 714–720. 10.1016/j.micinf.2007.02.015 17400016

[B155] van der Mee-Marquet N. Fourny L. Arnault L. Domelier A. S. Salloum M. Lartigue M. F. (2008). Molecular characterization of human-colonizing *Streptococcus agalactiae* strains isolated from throat, skin, anal margin, and genital body sites. *J. Clin. Microbiol.* 46 2906–2911. 10.1128/jcm.00421-08 18632904 PMC2546740

[B156] Verani J. R. McGee L. Schrag S. J. (2010). Prevention of perinatal group B streptococcal disease–revised guidelines from CDC, 2010. *MMWR Recomm. Rep.* 59 1–36. 21088663

[B157] Wilkinson H. W. Eagon R. G. (1971). Type-specific antigens of group B type Ic streptococci. *Infect. Immun.* 4 596–604. 5005310 10.1128/iai.4.5.596-604.1971 PMC416359

[B158] Xia F. D. Mallet A. Caliot E. Gao C. Trieu-Cuot P. Dramsi S. (2015). Capsular polysaccharide of Group B *Streptococcus* mediates biofilm formation in the presence of human plasma. *Microbes Infect.* 17 71–76. 10.1016/j.micinf.2014.10.007 25448634

[B159] Yang Y. Liu Y. Ding Y. Yi L. Ma Z. Fan H. (2013). Molecular characterization of *Streptococcus agalactiae* isolated from bovine mastitis in Eastern China. *PLoS One* 8:e67755. 10.1371/journal.pone.0067755 23874442 PMC3707890

[B160] Zawaneh S. M. Ayoub E. M. Baer H. Cruz A. C. Spellacy W. N. (1979). Factors influencing adherence of group B streptococci to human vaginal epithelial cells. *Infect. Immun.* 26 441–447. 44701 10.1128/iai.26.2.441-447.1979 PMC414634

[B161] Zimmermann P. Gwee A. Curtis N. (2017). The controversial role of breast milk in GBS late-onset disease. *J. Infect.* 74(Suppl. 1), S34–S40. 10.1016/s0163-4453(17)30189-5 28646960

